# Considering the Potential Health Impacts of Electric Scooters: An Analysis of User Reported Behaviors in Provo, Utah

**DOI:** 10.3390/ijerph17176344

**Published:** 2020-08-31

**Authors:** Jeffrey Glenn, Madeline Bluth, Mannon Christianson, Jaymie Pressley, Austin Taylor, Gregory S. Macfarlane, Robert A. Chaney

**Affiliations:** 1 Department of Public Health, College of Life Sciences, Brigham Young University, Provo, UT 84602, USA; mbluth13@byu.edu (M.B.); mannonlc@byu.edu (M.C.); jpressl3@byu.edu (J.P.); rchaney@byu.edu (R.A.C.); 2 Community and Neighborhood Services Department, City of Provo, Provo, UT 84601, USA; ataylor@provo.org; 3 Department of Civil and Environmental Engineering, Ira A. Fulton College of Engineering, Brigham Young University, Provo, UT 84602, USA; gregmacfarlane@byu.edu

**Keywords:** electric scooters, urban transport, public health

## Abstract

Electric scooters (e-scooters) are an increasingly popular form of transportation in urban areas. While research on this topic has focused primarily on injuries, there are multiple mechanisms by which e-scooter share programs may impact health. The aim of this study is to explore the health-related behaviors of e-scooter users and to discuss their implications for public health. Data were collected using an online survey emailed to registered e-scooter users. A total of 1070 users completed the survey. Descriptive variable statistics and chi-squared analysis were performed to determine variable dependent relationships and equality of proportions. The most common destinations reported were “just riding around for fun”, home, and dining/shopping. The two most common modes of transportation that would have been used if e-scooters were not available were walking (43.5%) and using a personal vehicle (28.5%). Riding behavior was equally mixed between on the street, on the sidewalk, and equal amounts of both. e-Scooters in Provo are likely having both positive (e.g., air pollution) and negative impacts on health (e.g., injuries, physical inactivity). Future research should further explore patterns of e-scooter use and explicitly examine the linkages between e-scooters and areas of health beyond just injuries.

## 1. Introduction

There is growing awareness in academic and policy circles of the close linkages between health and urban transportation practices [1]. Stand-up electric scooters (e-scooters), two-wheeled vehicles with a small electric motor and a thin deck on which a single rider stands, are a relatively new micro-mobility option for urban areas and have the potential for both positive and negative health impacts [2,3,4]. Although research on the health impacts of e-scooters is sparse, the topic merits further exploration given the rapid increase in e-scooter popularity over the past three years in the United States and around the world [5,6,7,8].

Gaining a better understanding of the true positive and negative health impacts of e-scooters must start with more fully understanding e-scooter users and patterns of use [9]. The potential health impacts of e-scooters depend on answers to questions related to user behaviors—e.g., substituting other forms of transit, commuting vs. recreational use, compliance with safety regulations. While some information exists to help answer these and other key questions, important knowledge gaps remain.

The aim of this study is to explore the health-related behaviors of e-scooter users in Provo, Utah four months after an e-scooter share program was introduced. Among the many evidence gaps that remain, this study focuses on four primary research questions: (1) What motivations do users have for riding e-scooters?; (2) What are the primary destinations of e-scooter users?; (3) What alternative travel mode would riders be using if not riding an e-scooter?; (4) To what degree are e-scooter users aware of and complying with safety regulations? (5) What program or policy changes do e-scooter users believe would improve Provo’s e-scooter share program? Based on this research, we identify opportunities for policy change that will facilitate positive health impacts of e-scooter use in Provo and other cities. We also hope to encourage researchers and policymakers to seek a deeper understanding of patterns of use in diverse contexts as they consider the broad range of potential health impacts of e-scooters.

### 1.1. Background: e-Scooter Share Programs

e-Scooter share programs were first introduced in the United States in Santa Monica, California in September 2017 and are now present in over 80 cities and 26 states throughout the country [5,10,11,12,13]. In 2018, users took 38.5 million trips on shared e-scooters in the United States [14]. Two of the largest e-scooter companies, Bird and Lime, were recently valued at over $2 billion each [6,15,16,17]. Multiple other companies, including ride share giants Uber and Lyft, have entered the competitive e-scooter market, which is predicted to become a $42 billion industry by 2030, although there is some evidence that the COVID-19 pandemic has contributed to reduced ridership numbers in recent months [18,19,20].

While there are variations between programs, in a typical e-scooter share arrangement a private company enters an agreement with local government officials to place e-scooters on city streets and make them available to rent for short periods of time [2,21,22,23]. Potential users download a mobile phone application that allows them to view the locations of available e-scooters in real time and to begin, end, and pay for their rides. Users are typically charged a flat fee for the rental plus an additional fee for each minute the e-scooter is used. Users leave their e-scooters at their final destinations where the e-scooters then become available to other users. Within municipal share programs, e-scooters typically have a range between 15 and 20 miles, and speeds are usually capped at 15 miles per hour [10].

e-Scooters are appealing for a variety of reasons. For users, e-scooters offer a convenient, affordable, fun transportation option that serves as an alternative to motor vehicles, biking, and walking [3,11]. e-Scooters are frequently used for both commuting and recreational purposes [6]. For local governments, e-scooters represent a new form of transportation that can help bridge the “last mile” gap, a common obstacle for transit use, by connecting people with public transit nodes [9,22,24,25]. e-Scooters are also seen as an environmentally friendly means for reducing traffic congestion in urban areas [26,27]. Moreover, e-scooter programs may be appealing to local officials because government funds are not usually required to start or maintain them; rather, e-scooter companies pay fees that allow government agencies to make infrastructure improvements for e-scooter riders [14]. e-Scooters may even be a contributing factor to economic development because they facilitate easier access to businesses located in urban centers where parking is scarce and motor vehicle travel is more difficult.

e-Scooters have been warmly welcomed by some municipalities and shunned by others as state and local governments have struggled to enact appropriate regulations to manage the rapid expansion of e-scooter share programs [5,21,28,29,30]. Significant variation in e-scooter laws exists between states and cities—e.g., helmet use, sidewalk riding, hours of operation. [6,10,21,28,31,32]. Since many state legislatures have not specifically addressed e-scooter usage, local governments have taken on the brunt of regulatory responsibility by attempting to manage e-scooter use with city ordinances [21]. e-Scooters create complicated liability issues in which municipalities may become liable for e-scooter injuries [10,21,31].

### 1.2. Background: e-Scooters and Health

**Figure 1:**
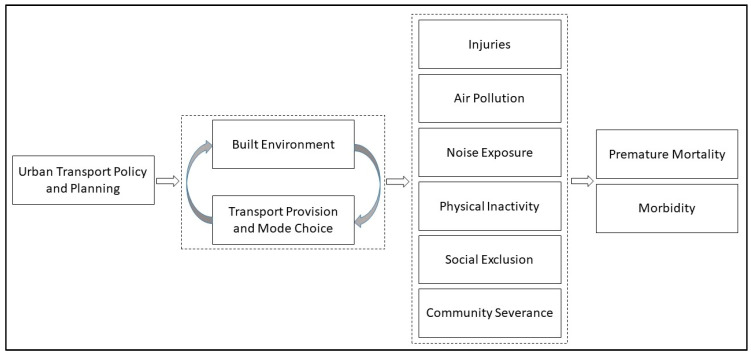
Linkages between e-scooters and health. Adapted from Khreis et al., 2017 [1].

There is a range of mechanisms through which e-scooters may affect health. In a recent evidence review, Khreis et al. found multiple linkages between urban transport exposures or practices and adverse health impacts [1]. While the research on e-scooters and health is limited, many of these linkages have been shown or theorized to apply to e-scooters discussed below. Figure 1 highlights these linkages and illustrates that they are shaped by available transport options and features of the built environment.

#### 1.2.1. Injuries

A primary public health concern, and the focus of the vast majority of academic research on e-scooters to date, is e-scooter-related injuries [10]. Several studies in the United States and elsewhere have found a high incidence of injuries related to scooter usage, particularly head and limb trauma, after the introduction of e-scooter share programs [5,12,24,28,33,34,35]. There is even some evidence that the injury rate for e-scooters may be higher than that of motorcycles and personal vehicles [27,36]. Most injuries are due to falls or collisions with objects (not with motor vehicles) that occur due to poor road conditions or excessive speeds [5,23,33,35,37]. There have also been reports of burns resulting from explosions of batteries [35,38]. In the United States at least nine known deaths have been linked with e-scooter use [10].

Various factors contribute to the prevalence of e-scooter injuries: Incompatible infrastructure (e.g., lack of bike lanes), lack of directional tools on e-scooters (e.g., turn signals, headlights), rider inexperience and noncompliance with age restrictions, failure of users to obey traffic rules, alcohol use, and reluctance to wear helmets [5,7,24,28,33]. Recent studies have found helmet use among injured e-scooter riders to be extremely low, ranging from 0% and 8% in most studies [5,6,23,24,28,34,35,39]. Additionally, despite regulations prohibiting them from doing so, e-scooter users commonly ride and park on sidewalks, which can lead to injuries to users as well as to pedestrians [40,41]. One study found that 44% of collisions occurred on sidewalks where riding was prohibited, and others have found that approximately 10% of all e-scooter related injuries involve pedestrians [5,7,10,23,26,31,33,42]. Vulnerable populations such as the elderly, hearing impaired, and young children have an increased risk for sidewalk-related injuries [31]. A sizeable proportion of e-scooter injuries among users involve children under 18, despite most rental company agreements prohibiting ridership for minors [5]. Many of these hazards relate to cultural norms and limited regulation that may minimize users’ perception of potential dangers and therefore lead to unsafe behaviors [28].

#### 1.2.2. Environment (Air Pollution/Noise Exposure)

In providing an electric alternative to motor vehicles, e-scooters are typically perceived as an environmentally friendly form of transportation that could lead to lower vehicle emissions and cleaner air in cities where they are being used [3]. Since air pollution is responsible for premature morbidity and mortality from a number of diseases—including, for example, respiratory infections, cardiovascular disease, and premature birth—the potential positive impact of e-scooter use on health is significant [1,43]. Exposure to noise from motor vehicle engines, which has been linked to increased incidence of ischemic heart disease, cognitive impairment among children, and sleep disturbance, is also inversely correlated with e-scooter use since the battery-operated engines are essentially silent [1,10].

Although e-scooters are commonly seen as a green alternative to gasoline-powered motor vehicles, they present a number of environmental concerns—including greenhouse gas emissions, particulate matter formation, and use of mineral and fossil resources—that often go overlooked [44,45,46,47]. Findings from recent studies suggest that, overall, e-scooters have a more negative life cycle impact on the environment than the transportation modes they are replacing [2,47]. One study found that e-scooters’ impact on climate change is better than that of personal automobiles but worse than that of buses with higher ridership or electric bicycles [2]. With an average lifespan of about 2 years before often ending up in landfills, e-scooters’ high level of disposability is a key driver of negative environmental impacts, which means that this impact will likely lessen as technology improves, a goal e-scooter companies are actively workings towards [2,47,48]. Another major issue is the vehicles used to collect e-scooters each day for charging and relocating. One study found that the 43% of the emissions attributable to e-scooters stem from collection vehicles [2]. More sparsely populated areas likely necessitate higher collection miles driven and thus the e-scooters will likely lead to more air pollution than in densely populated urban areas. Another environmental concern is the greenhouse gas emissions required to manufacture and assemble e-scooters [2]. This process includes the extraction of raw materials, including aluminum and lithium, for the e-scooter frames and batteries.

#### 1.2.3. Physical Inactivity

Very little research exists on the linkage between e-scooters and physical activity. Insufficient physical activity is responsible for over 2 million deaths each year as a key risk factor for multiple chronic diseases [49]. Because the act of riding e-scooters in itself likely offers few physical activity benefits, some health researchers have expressed concerns that e-scooters will replace active forms of transportation such as walking and cycling [50,51,52]. On the other hand, some advocates have observed a positive association between the increase in e-scooters and more active transportation as cities seeking to accommodate e-scooters have improved infrastructure that indirectly creates an environment and culture more conducive to cycling and walking [53,54]. Some e-scooter companies have argued that e-scooters offer a low-intensity workout that can help users increase core strength and exercise their legs, in addition to acting as a “gateway activity” to further exercise [55]. While these specific claims have yet to be confirmed through research, some preliminary conclusions that e-scooters offer the potential for at least minor physical activity benefits may be drawn from the literature about the positive health benefits of standing compared to sitting [56,57,58].

#### 1.2.4. Social Exclusion and Community Severance

Critical questions remain regarding the effects of e-scooters share programs on social life in communities in which they operate. Community health and social interaction, which are influenced by neighborhood design and transport infrastructure, have a significant impact on mental health and well-being of community members [1,59,60]. Community severance occurs where transportation acts as a physical or psychological barrier that separates built-up areas or open spaces [1]. There are reasons to believe e-scooters may increase community connectedness by improving access to transit, recreation facilities, and other public spaces where social interaction occurs. On the other hand, e-scooters may also contribute to community severance, for example by increasing risks of pedestrian injuries or by acting as a visual symbol of disorder in urban neighborhoods due to erratic placement of e-scooters after use [50,61]. There are reports of frustrated city residents vandalizing e-scooters and even celebrating their actions by posting evidence of that vandalism on social media [62]. Even if e-scooters do not represent a new barrier to community connectedness, the benefits of e-scooter access may not be available equitably to people of lower socioeconomic statuses while any negative health impacts may disproportionately affect these same people, which could exacerbate existing inequalities [51].

## 2. Methods

### 2.1. Study Context

**Figure 2:**
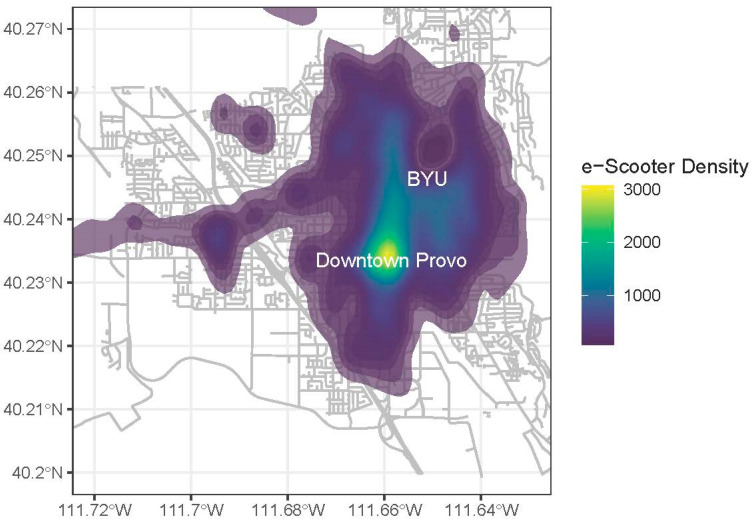
Density of e-scooter trip points in October 2019. Data from Zagster via Provo City, background streets supplied by OpenStreetMap.

This study looked at e-scooter rider behavior in Provo, UT, a city of 116,000 people [63] located approximately 40 miles south of Salt Lake City, UT. The close proximity of two large universities within or near Provo City limits has contributed to a high concentration of residents and traffic. Provo’s mayor was primarily interested in the e-scooter program to improve the air quality of the city by providing zero emission alternatives to driving [64]. In partnership with the company Zagster, an e-scooter share program was introduced in Provo in August 2019. The geographic area principally targeted in the e-scooter program lies between downtown Provo and Brigham Young University (BYU), where there is a high concentration of college-aged residents, relatively dense commercial and educational land use, and a new Bus Rapid Transit (BRT) line. BYU does not permit e-scooters on campus. Figure 2 shows the geospatial distribution of e-scooter rides observed in October 2019. At the time of the survey, 500 total e-scooters were available on city streets. Between August 19 and December 31, over 85,000 rides were taken on Provo’s e-scooters [65]. Provo City Code 9.15.200 prohibits e-scooter use on sidewalks [66] Helmet use is not required but is strongly encouraged. While Utah state law prohibits people under eight years old from riding an e-scooter, Zagster policy requires users to be eighteen or older to rent an e-scooter.

### 2.2. Study Design

This study was a cross sectional study designed to address the primary research questions. Data were collected using a 13-item online questionnaire; three were demographic questions and 8 questions were about riding history, behavior, and knowledge (see Appendix A for full question list). Demographics included city residence, age, and gender. Riding behavior questions included trip origin, trip destination, trip motivation, and street versus sidewalk riding on users’ most recent e-scooter trip. Open-ended responses were solicited related to changes that would enable street versus sidewalk riding and to e-scooter staging. The survey was emailed the week of 24 September 2019 to all registered Zagster users (~15,000) in Provo City. A total of 1070 users completed the survey, for a response rate of 7.1%. All research procedures were performed in compliance with relevant laws and institutional guidelines.

### 2.3. Data Analysis

Participant demographic characteristics (age, gender, and place of residence) were first calculated, and descriptive variable statistics were then conducted for each item in the questionnaire. After verifying statistical assumptions, chi-squared analyses were performed to determine variable dependent relationships and equality of proportions between demographic characteristics and motivations for riding, destinations, travel mode alternatives, and safety behaviors. Quantitative analysis was performed using the R statistical software (R Foundation for Statistical Computing, Vienna, Austria) [67]. Two researchers used NVivo qualitative data analysis software to thematically code responses to the open-ended survey questions [68]. These coded responses were then analyzed collectively by the full research team to identify the most prominent emergent themes.

## 3. Results

The majority of respondents were 18–24 years old (56.2%), and 5% were under 18 years old. More men than women completed the survey (63% vs. 37%). Roughly 95% of participants were residents of Utah County (Provo City—85.0%, Utah County—11.9%) (Table 1).

### 3.1. Motivations for Riding e-Scooters

The most frequently mentioned reason for riding e-scooters was “to have fun” (42.2%) followed by “to save time” (32.3%) (see Table 2). Though “having fun” was the top reason for riding e-scooters for both men and women, significantly more women (48.3%) reported riding for this reason compared to men (39.1%) (χ^2^ = 12.3, df = 1, *p* < 0.001). Similarly, men were more likely to ride “to avoid parking hassles” (14.8%) compared to women (9.0%) (χ^2^ = 10.3, df = 1, *p* = 0.001).

College-aged (CA) persons aged 18–24 years old comprise the largest portion of e-scooter ridership in Provo City (56.2%). While more non-CA persons (49.4%) than CA persons (37.6%) reported a motivation for riding e-scooters was to have fun (χ^2^ = 21.35, df = 1, *p* < 0.001), more CA than non-CA persons reported a motivation was to save time (39.2% compared to 22.4%; χ^2^ = 48.73, df=1, *p* < 0.001).

### 3.2. Destinations of e-Scooter Riders

The most common destinations to which e-scooters are reportedly being ridden are “just riding around for fun” (25.3%), home (20.0%), and dinning/shopping locations (17.1%) (see Table 3, a full table is presented in Appendix A). There were no statistical gender differences with respect to destination with the exception of school; men tended to ride to school more (13.2%) than women (8.2%) (χ^2^ = 5.64, df = 1, *p* = 0.02).

### 3.3. Travel Mode Alternatives if Not Using an e-Scooter

The two most common modes of transportation that would have been used if e-scooters were not available were walking (43.5%) and using a personal vehicle (28.5%) (see Table 4). The only statistical difference by gender was for bicycling, where men were more likely to use a bicycle if an e-scooter were unavailable (5.2% vs. 1.9%) (χ^2^ = 6.16, df = 1, *p* = 0.01).

### 3.4. Awareness of and Compliance with Safety Regulations

Riding behavior was equally mixed between on the street (n = 369, 34.6%), on the sidewalk (n = 357, 33.4%), and equal amounts of both (n = 342, 32.0%). Sidewalk and street riding was associated with gender in that men were more likely to ride on the street (χ^2^ = 11.01, df = 1, *p* < 0.001) and women were more likely to ride on the sidewalk (χ^2^ = 3.01, df = 1, *p* = 0.08). There was no difference between genders who reported to ride equally on the street and sidewalk. Likewise, CA persons were less likely to ride on the street (30.2% vs. 40.2%; χ^2^ = 11.20, df = 1, *p* < 0.001) and more likely to ride on the sidewalk (38.3% vs. 27.0%; χ^2^ = 14.6, df = 1, *p* < 0.001). There was no difference by age for those who equally rode between sidewalk and street. The majority of respondents did not know that it is illegal, according to Provo City code, to ride e-scooters on the sidewalk (n = 691, 64.7%). There were no differences between genders but there were by age. College-aged persons were less likely to know about the sidewalk riding code (31.6% vs. 40.6%; χ^2^ = 9.06, df = 1, *p* = 0.002).

### 3.5. Changes to Enable Safer On-Street (vs. Sidewalk) Riding

When asked what program changes would make them ride on the street rather than the sidewalk, some participants (12%) reported that they would have ridden in the street if they had known that it was acceptable to do so and/or they could be sure drivers were aware that they were allowed to do so. Overwhelmingly, most of the respondents (74%) asked for the addition of bike lanes and/or better constructed bike lanes throughout Provo. There were very few mentions of where the bike lanes should be added. Most respondents use the adjectives “good”, “wider”, “improved”, “clearly marked”, and “painted” when describing what was meant by better bike lanes. The next highest response called for better roads; 16% of respondents said that Provo streets had potholes, narrow roads, bumpy streets, and a lack of lane divisions. Reckless drivers and curbside parking—which blocks bike lanes, takes up room on the shoulders, and pushes scooter riders further into the center of the road—were cited as deterrents to riding off the sidewalk. When asked if there was anything else users wanted to mention about the scooters, many of them (15%) simply stated that they enjoyed having scooters in the area.

## 4. Discussion

This study sought to consider the relationship between e-scooters and health by gaining a better understanding of e-scooter users and their behaviors in Provo, UT. While e-scooters may affect health in the various ways, whether they have a net positive or negative impact on health depends largely on why and how people are riding them. Two-thirds of users who responded to the survey were men, and over half were 18–24 years old. This age range is similar to what we would expect given the age demographics of Provo where the median age is 23.6 and 44% of the population is between ages 20–29 [63]. While injury data from Provo has not yet been reported, previous studies in other cities found that the majority of e-scooter injuries were among male millennials, the same demographic group who make up the majority of e-scooter users in Provo [6,12,37,69].

User compliance with safety regulations is another important health-related factor addressed by these data. Despite the Zagster policy requiring renters to be at least 18 years of age, 5% of all respondents were under 18, which raises concerns about user safety and the ability of Zagster (and other private e-scooter companies) to enforce its rider policies. Additionally, only 34.6% of users reported complying with local law and riding exclusively on the street while the rest reported riding at least partially on the sidewalk. While data from other cities is limited, a pilot e-scooter program in Portland, OR found that the proportion of sidewalk riders varied greatly depending on street design—18% rode on sidewalks with a 20 mph speed limit compared to 66% with a 35 mph speed limit, and 8% rode on sidewalks if a protected bike lane existed compared to 39% where there were no bike facilities [42]. Riding on sidewalks is overall more dangerous for users and much more likely to lead to pedestrian injuries as have been found in previous studies [3,10,31]. The finding that women were more likely than men to avoid on-street riding is consistent with research on gender differences in cycling behavior that finds safety perception is a major factor [70,71]. The majority of users (64.7%) reported being unaware that e-scooters were not permitted on sidewalks, which represents a higher proportion of riders compared to the 43% of users in Rosslyn, VA who were not familiar with e-scooter laws concerning sidewalk riding [40]. While the difference between cities likely reflects that the e-scooter program in Rosslyn had been active for a longer period of time, the lack of knowledge around laws suggests that better educating users may be a first step in reducing unsafe riding behavior. Nearly 75% of users in Provo mentioned in their open-ended responses that additional and improved bike lanes would make it easier for them to ride on the street, which highlights another opportunity, albeit one requiring a greater financial investment from the city, to create a safer environment for e-scooter users.

The data show that e-scooter users in Provo choose to ride for a variety of reasons. The top reason given was to have fun (42.2%) and the top destination reported by users was “just riding around for fun” (25.3%). However, a sizeable number of users also report riding e-scooters to commute to work (7.9%) or school (11.4%) and for other purposes such as dining/shopping (17.1%) and traveling to social gatherings (16%). These numbers are similar to those in Portland, OR where 28.6% of riders used e-scooters for recreation or exercise while 71% used them to get to a destination [42]. Convenience appears to be an important motivator as the second and third top reasons given for riding e-scooters in Provo were to save time (32.3%) and to avoid parking hassles (12.9%). Interestingly, CA users were more likely to rent e-scooters to save time than to have fun whereas non-CA users reported the opposite. CA users were also more likely to ride to school and social gatherings while non-CA users were more likely to ride to dine out/shop or commute to work. Our findings suggest that age is more influential on trip destination as opposed to gender. A very small percentage of riders (2.3%) reported their destination as a public transit stop, which may indicate that e-scooters in Provo are not necessarily delivering on the promise of solving the “last mile” problem, although because the questionnaire asked specifically about the most recent trip it is likely that some of the other riders were connecting *from* public transit. This is key question that should be addressed through a different survey design in future research.

Given the variety of motivations cited for riding e-scooters, a critical question in terms of health implications is: For which alternative modes of travel are e-scooters being substituted? The most common response, given by 43.5% of users, was that they would have walked if an e-scooter had not been available; among CA riders this percentage increased to 50.4%. Additionally, 4% of users reported riding e-scooters instead of bicycling. Similarly, in Portland, OR 37% and 5% of e-scooter riders, respectively, would have walked or biked instead of using an e-scooter, and in Raleigh, NC 49% of riders would have walked or biked [2,42]. The most likely impact of these findings is an overall reduction in physical activity levels because e-scooters are replacing more active forms of transportation. While this may be cause for concern in terms of health, on the other hand, 29.4% of e-scooter users reported that they would have used a personal vehicle or rideshare service (i.e., Uber, taxi) if an e-scooter had not been available. This number is comparable to data from Rosslyn, VA (39%), Raleigh, NC (34%), and Portland, OR (34%) [2,40,42]. (In Portland, 6% of users even reported getting rid of a personal vehicle due to e-scooter availability [42].) These rides represent fewer cars on the road and, in all likelihood, an overall reduction in local air pollution and associated poor health. Additionally, the survey does not capture the possibility that the respondent would have chosen a different destination entirely were an e-scooter not available. Given that e-scooters are best designed for short trips in urban areas, it is possible that the avoided motor vehicle trips would have been longer than their e-scooter substitutes. This finding is particularly relevant for Provo City, a place with problematic winter air pollution and whose primary motivation for introducing e-scooters was to provide a green alternative to motor vehicles; yet, considering disposability issues and emissions due to collecting and placement of e-scooters, important questions remain about the full environmental impact and its implications for health.

Based on the findings of this study, there are several policy change strategies that could help optimize the heath impacts of e-scooter share programs in Provo and in other cities. First, to reduce the probability of injuries, more training and strategically placed educational information (e.g., signs posted in high traffic areas) should be provided to increase users’ knowledge about safety precautions (e.g., avoiding sidewalks, safe parking) and users’ e-scooter operating skills. Considering the shared road space, information should also be provided to help drivers, cyclists, and pedestrians be more aware of e-scooter riders.

Second, as evidenced by ridership among children, there is an enforcement gap in Zagster’s ability to enforce safety policies. Similarly, although this study did not explicitly explore helmet use, informal observation on the streets of Provo suggest that helmet use is extremely rare among e-scooter users, which is consistent with other studies [7,10,33]. To improve safety, cities should work with private e-scooter companies to identify ways, which may include the passing of additional local ordinances, to identify violations and enforce policies.

Third, for e-scooters to experience long term success it is clear that bike lines and other infrastructure must continue to improve. When asked about possible improvements that would encourage them to ride on the street, the vast majority stated designated lanes would be most helpful (n = 787). Enhanced education and training alone will likely be ineffective without a more conducive riding environment, which should be a priority for city decision makers concerned with improving safety

Fourth, while a sizeable proportion of users are substituting e-scooters for personal vehicles, there are still negative environmental impacts that should be considered and minimized. Zagster recently introduced a new, more durable model of e-scooter to Provo City streets, and city policymakers should continue to push for e-scooters that have longer durability. They should also work with Zagster to ensure low-emissions vehicles are used for collecting and placing e-scooters, and that the routes driven for these tasks are as short as possible.

Finally, cities should consult regularly with community members—those who use e-scooters and those who do not—to understand the impacts of e-scooters on community severance and social interactions, particularly among marginalized populations.

This study makes a significant contribution to the literature by applying an existing health impact framework and proposing a range of linkages between health and e-scooters, a rapidly emerging public health issue for which previous studies have focused almost exclusively on injuries. The study also reports data from a relatively large sample of e-scooter users on their self-reported behavior, which is scarce in the academic literature, that serve as starting point for understanding how population health may be impacted by e-scooters. These data led to concrete, valuable recommendations for policymakers in Provo and other places, especially mid-sized cities, which are currently grappling with instituting appropriate policy responses for the variety of issues that come with e-scooter programs. Some critical limitations should be noted. First and foremost, while this study considered linkages between e-scooters and health, its findings do not directly address the health impact of e-scooters on Provo residents. Even where it adds value in terms of providing a clearer picture of why and how e-scooters are being used, it omits key demographic variables (e.g., race/ethnicity, income, etc.) that are essential for understanding some of the connections between e-scooters and health (e.g., community severance). Finally, the survey used is potentially problematic because it represents a single point in time shortly after e-scooters were introduced in Provo. It also asks only about users’ most recent trip and it relies on responses from a small, non-representative sample of registered Zagster users, which may be a source of bias if those who elected to respond to the survey have different patterns of behavior than those who did not respond. The use of an online survey may also lead to bias by selecting for younger, internet-using adults; however, this is not a major concern because these respondents are also the most likely to be e-scooter users.

## 5. Conclusions

e-Scooters are a nascent public health issue that positively and negatively affect health in a number of ways, including through injuries, air pollution levels, physical activity levels, and community severance. To understand the full impact of e-scooters on health we need to gain a thorough understanding of e-scooter users and their patterns of use. This study found that in Provo, UT e-scooter users are predominantly male, college-aged individuals who ride e-scooters for a variety of reasons, the top being for recreational purposes. Most users were unaware of laws prohibiting e-scooters from sidewalk riding, which led to two-thirds of users riding at least part of the time on sidewalks. About half of users would be walking or riding bicycles if e-scooters were not an option, while about one-third would be driving a personal vehicle. Thus, e-scooters in Provo are likely having both positive and negative impacts on health. Future research, perhaps in the form of a health impact assessment, should be designed explicitly to examine the linkages between e-scooters and areas of health beyond just injuries, e.g., by focusing on community severance among marginalized communities or on users’ physical activity levels. Research is also needed to evaluate the impact of policies and interventions designed to reduce e-scooter related injuries. Thoughtful, evidence-based implementation of e-scooter programs is critical to ensuring a future net positive benefit to public and community health.

## Figures and Tables

**Table 1:** Participant demographics from e-scooter survey.

Age (Years)	Total Frequency (%)	Male	Female
Under 18	53 (5%)	33 (4.9%)	19 (4.9%)
18–24	601 (56.2%)	357 (53.0%)	242 (62.1%)
25–34	212 (19.8%)	141 (20.9%)	71 (18.2%)
35–44	114 (10.7%)	83 (12.3%)	29 (7.4%)
45–54	62 (5.8%)	40 (5.9%)	22 (5.6%)
55–64	24 (2.2%)	17 (2.5%)	7 (1.8%)
65+	4 (0.04%)	3 (0.4%)	0 (0.0%)
Total participants	1070	674 (63.0%)	390 (37%)

**Table 2:** Motivations for riding e-scooters.

Reason	Total Count (%)	Male	Female	College-Age	Non-College-Age
To have fun	669 (42.2%)	401 (39.1%)	268 (48.4%)	345 (37.6%)	324 (49.4%)
To save time	512 (32.3%)	338 (32.9%)	174 (31.4%)	360 (39.2%)	147 (22.4%)
To avoid parking hassles	205 (12.9%)	152 (14.8%)	50 (9.0%)	120 (13.1%)	80 (12.2%)
For environmental reasons	86 (5.4%)	57 (5.6%)	26 (4.7%)	34 (3.7%)	48 (7.3%)
Other	58 (3.66%)	44 (4.3%)	16 (2.9%)	24 (2.6%)	38 (5.8%)
To save money	54 (3.4%)	34 (3.3%)	20 (3.6%)	35 (3.8%)	19 (2.9%)
Total	*n* = 1584	1026	554	918	656

Note: This was a select-all-that-apply question.

**Table 3:** Where e-scooters are being ridden by gender.

Destination	Total Count (%)	Male	Female	College-Age	Non-College-Age
Rode for fun	269 (25.3%)	151 (22.4%)	118 (30.3%)	116 (19.4%)	153 (32.9%)
Home	213 (20.0%)	138 (20.5%)	75 (19.2%)	141 (23.5%)	72 (15.5%)
Dining/shopping	182 (17.1%)	112 (16.6%)	70 (17.9%)	92 (15.4%)	90 (19.4%)
Social gathering	170 (16.0%)	107 (15.9%)	63 (16.2%)	104 (17.4%)	66 (14.2%)
School	121 (11.4%)	89 (13.2%)	32 (8.2%)	102 (17.0% )	19 (4.1%)
Work	84 (7.9%)	58 (12.9%)	26 (6.7)%	33 (5.5%)	51 (11.0%)
Public transit stop	25 (2.3%)	19 (2.8%)	6 (1.5%)	11 (1.8%)	14 (3.0%)
Total	n = 1064	n = 674	n = 390	599	465

The destinations of CA persons were different in many instances compared with non-college-aged persons. CA persons were less likely to use the e-scooter when dining out/shopping (χ^2^ = 2.67, df = 1, *p* = 0.10; CA = 15% vs. non-CA = 24%); to just ride around for fun (χ^2^ = 24.67, df = 1, *p* < 0.001; CA = 19.4% vs. CA = 32.9%); and, to work (χ^2^ = 9.99, df = 1, *p* = 0.002; CA = 5.51% vs. non-CA = 10.97%). Conversely, they were more likely to ride home (χ^2^ = 10.11, df = 1, *p* = 0.001; CA = 24% vs. non-CA = 18%); to school (χ^2^ = 42.23, df = 1, *p* < 0.001; CA = 17.03% vs. non-CA = 4.09%); and to social gatherings (χ^2^ = 1.73, df = 1, *p* = 0.19; CA = 17.4% vs. non-CA = 14.2%).

**Table 4:** Reported travel mode alternative to e-scooters.

Alternative Mode	Total Count (%)	Male	Female	College-Age	Non-College Age
Bicycle	41 (4.0%)	34 (5.2%)	7 (1.9%)	17 (2.8%)	24 (5.5%)
Not taken trip	113 (10.9%)	50 (7.6%)	63 (16.7%)	52 (8.7%)	61 (14.1%)
Personal vehicle	294 (28.5%)	196 (29.9%)	98 (25.9%)	149 (24.9%)	145 (33.4%)
Pick up/drop off	32 (3.1%)	22 (3.4%)	10 (2.6%)	11 (1.8%)	21 (4.8%)
Public transit	88 (8.5%)	59 (9.0%)	29 (7.7%)	61 (10.2%)	27 (6.2%)
Rideshare	9 (0.9%)	4 (0.6%)	5 (1.3%)	4 (0.7%)	5 (1.2%)
Walking	449 (43.5%)	284 (43.4%)	165 (43.7%)	302 (50.4%)	147 (33.9%)
Other	7 (0.7%)	6 (0.9%)	1 (0.3%)	3 (0.5%)	4 (0.9%)
Total	1033	655	378	599	434

Similar to trip destination, there were significant differences between the 18–24-year-old CA and non-CA group. As an alternative to e-scooters, CA persons were less likely to have used a bicycle (χ^2^ = 4.10, df = 1, *p* = 0.04; CA = 2.84% vs. non-CA = 5.53%); to have not taken the trip (χ^2^ = 6.92, df = 1, *p* = 0.01; CA = 8.68% vs. non-CA = 14.06%); to use a personal vehicle (χ^2^ = 8.59, df = 1, *p* = 0.003; CA = 24.87% vs. non-CA = 33.41%), and to be picked up/dropped off (χ^2^ = 6.59, df = 1, *p* = 0.01; CA = 1.84% vs. non-CA = 4.84%). Conversely, CA persons were more likely to use public transportation (χ^2^ = 4.57, df = 1, *p* = 0.01; CA = 10.18% vs. non-CA = 6.22%) and to walk (χ^2^ = 27.37, df = 1, *p* < 0.01; CA = 50.42% vs. non-CA = 33.87%).

## Appendix A—Zagster Rider Survey Questions

1. On your last trip, why did you choose to ride a scooter?
2. On your last trip, what mode of transportation would you have taken had a scooter not been available?
3. On your last trip, where did you ride to?
4. On your last trip, where did you ride from?
5. On your last trip, where did you primarily ride?
6. If you rode on sidewalks, why did you choose to do so?
7. Did you know that Provo City Code 9.15.200 prohibits riding on sidewalks? (Don’t worry, we won’t tell on you.)
8. What changes would make you want to ride in the street instead of the sidewalk?
9. Where should scooters be staged in the morning that they aren’t currently?
10. Anything else you’d like to tell us?
11. What city do you live in?
12. What is your age?
13. What is your gender?
